# Supplementary material for: PD-L1 Dependent Immunogenic Landscape in Hot Lung Adenocarcinomas Identified by Transcriptome Analysis
Source: Cancers (Basel). 2021 Sep 11;13(18):4562. doi: 10.3390/cancers13184562 (PMC8469831; doi:10.3390/cancers13184562)
Supplement: Supplementary file 1 [file cancers-13-04562-s001.zip › cancers-1353667-supplementary.pdf]

cancers

MDPI

Supplementary Material: PD-L1 Dependent Immunogenic Landscape in Hot Lung Adenocarcinomas Identified by Transcriptome Analysis

Jutta Kirfel, Christiane Charlotte Kümpers, Anke Fähnrich, Carsten Heidel, Mladen Jokic, Ignacija Vlasic, Sebastian Marwitz, Torsten Goldmann, Helen Pasternack, Sabine Bohnet, Danny Jonigk, Mark P. Kühnel, Anne Offermann, Hauke Busch and Sven Perner

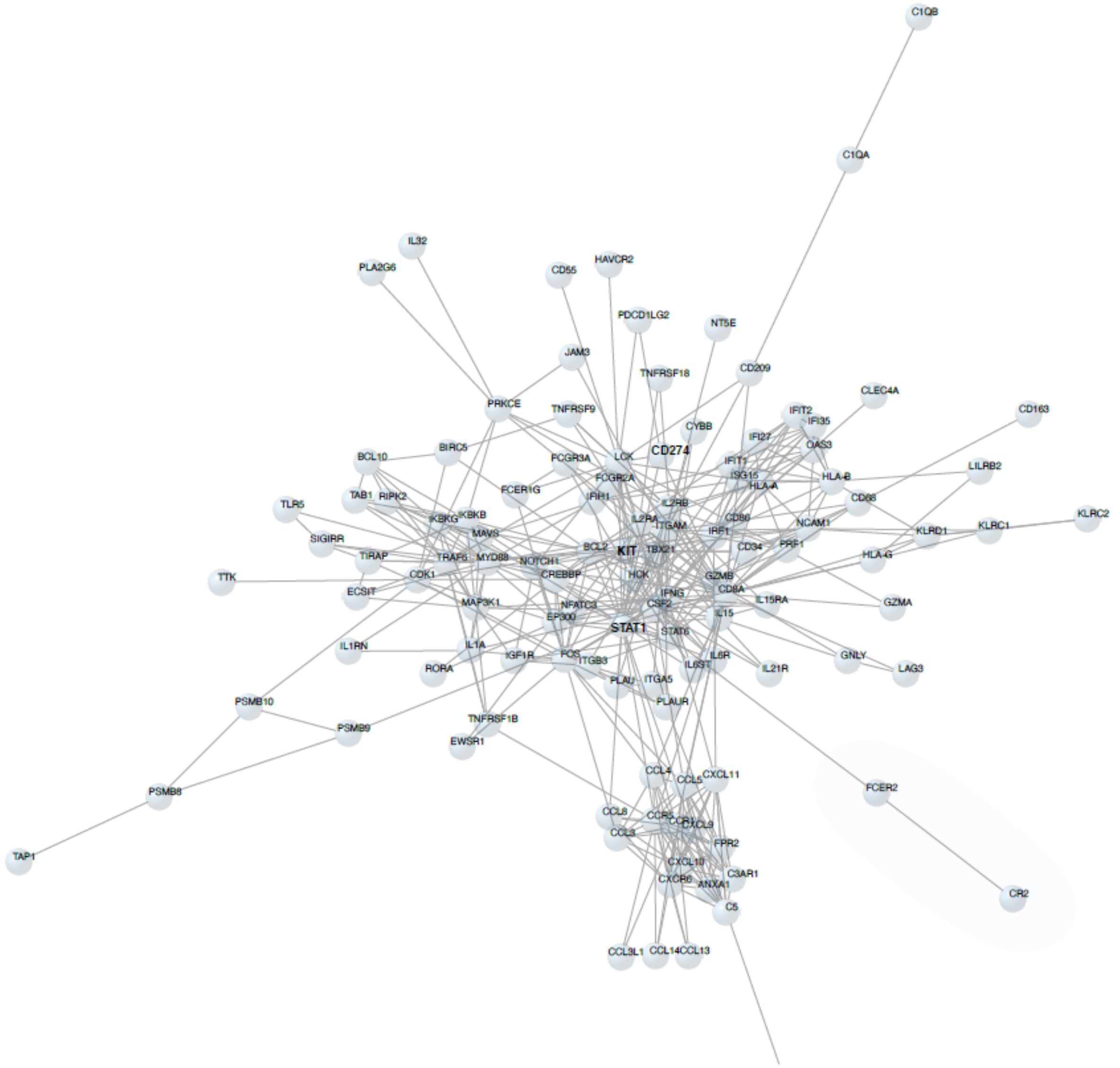

Figure S1. Centrality degree of the subnetwork Boxplot. showing Centrality Scores to evaluate the importance of the nodes of the network. The centrality degree of each gene was calculated using the function centralization.betweenness of the graph package (v1.2.5). The results were visualized as boxplot.

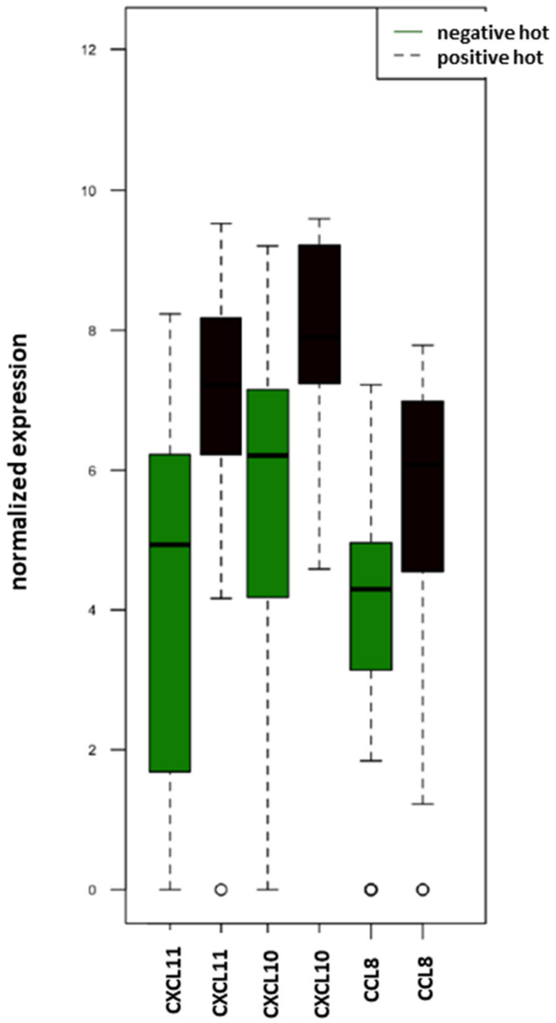

Figure S2. Boxplot of transcriptomic analysis showing over-expressed genes. Boxplot of transcriptomic analysis showing over-expressed genes CXCL11, CXCL10 and CCL8 in PH LUADs compared to NH counterparts encoding for chemokines associated with chemoattraction for T-cells and NK-cells.

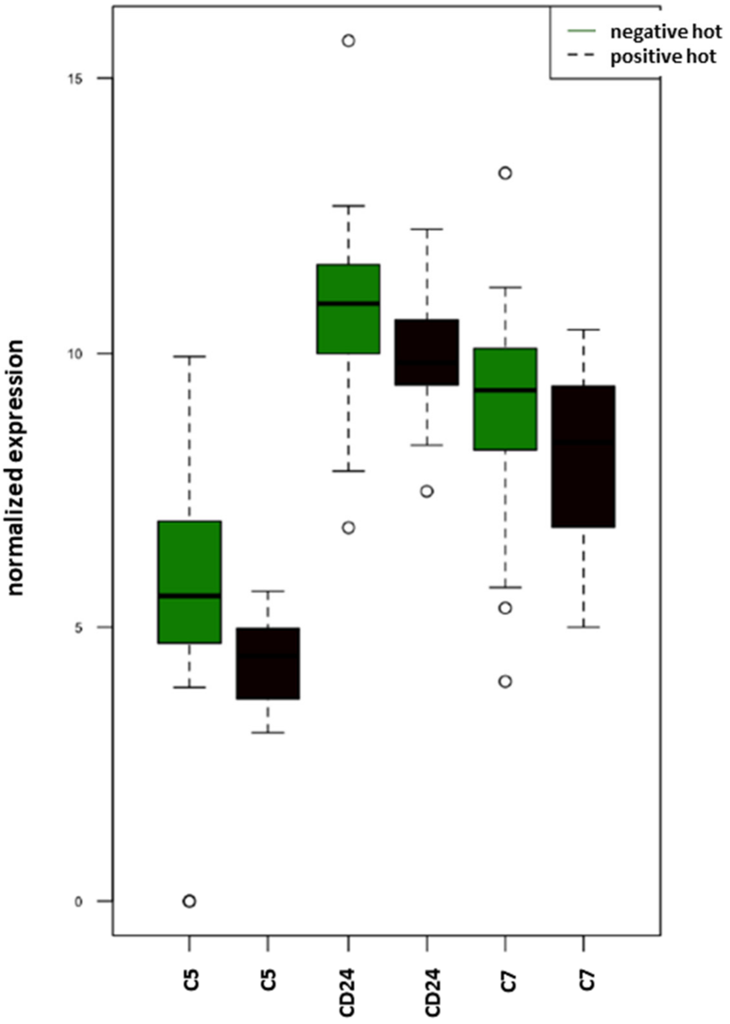

Figure S3. Boxplot of transcriptomic analysis showing down-regulated genes. Boxplot of transcriptomic analysis showing down-regulated genes C5, CD24 and C7 in PH LUADs compared to NH counterparts encoding for innate and adaptive immune response.

		TILs diffusely infiltrating tumor structures	TILs excluded from tumor structures	emphasis of TILs in the tumor margin	TILs not present	lymphfollicular structures at the margin	lymphfollicular structures intratumoral	CD8+ lymphocytes per area	CD4+ lymphocytes per area	CD8/CD4 quotient
**group**	**case**									
NC	1		x					5	5	1
NC	2		x					15	20	0.75
NC	3		x					5	10	0.5
NC	4		x			x		15	5	3
NC	5				x			2	2	1
NC	6		x			x		2	5	0.4
NC	7		x			x		5	5	1
NC	8				x			2	2	1
NC	9				x			2	5	0.4
NC	10		x					5	5	1
NC	11		x				x	15	20	0.75
NC	12		x			x	x	2	10	0.2
NC	13		x			x		20	15	1.3
NC	14		x					5	5	1
NC	15		x			x		10	15	0.67
NC	16		x					10	15	0.67
NC	17				x			2	2	1
NC	18		x					2	2	1
NC	19		x					5	10	0.5
NC	20		x	x				10	15	0.67
NC	21		x					5	10	0.5
NC	22		x					5	5	1
NC	23				x			2	5	0.4
NC	24		x					5	5	1
NC	25		x					10	10	1
NC	26		x				x	2	5	0.4
NC	27		x				x	5	15	0.3
NC	28			x		x		2	5	0.4
NC	29		x	x				10	10	1
NC	30				x			5	5	1
NC	31		x			x		10	5	2
NC	32		x				x	5	10	0.5
NC	33				x			15	20	0.75
NC	34		x			x		5	10	0.5
NC	35		x			x		5	5	1
NC	36		x			x		2	5	0.4
NC	37		x					5	10	0.5
NC	38		x			x		2	2	1
NC	39		x	x				10	15	0.67
NC	40		x					5	10	0.5
NC	41		x					5	10	0.5
NC	42		x				x	5	5	1
NC	43		x				x	5	10	0.5
NC	44				x			5	5	1
NC	45		x	x				5	10	0.5
NC	46		x	x				15	15	1
NC	47		x				x	15	20	0.75
NC	48				x			15	20	0.75
NC	49				x			2	5	0.4
NC	50				x			10	5	2
NC	51				x			5	15	0.33
NC	52		x					5	10	0.5
NC	53		x					10	20	0.5
NC	54		x	x				5	20	0.25
NC	55		x					10	10	1

**NH**	**1**		**x**	**x**		**x**		**20**	**25**	**0.8**
**NH**	**2**	**x**				**x**		**10**	**20**	**0.5**
**NH**	**3**		**x**	**x**		**x**		**15**	**25**	**0.6**
**NH**	**4**	**x**						**0.1**	**20**	**0.005**
**NH**	**5**		**x**	**x**		**x**		**10**	**10**	**1**
**NH**	**6**		**x**					**10**	**20**	**0.5**
**NH**	**7**	**x**								
**NH**	**8**	**x**						**20**	**20**	**1**
**NH**	**9**	**x**					**x**	**10**	**25**	**0.4**
**NH**	**10**	**x**					**x**	**10**	**20**	**0.5**
**NH**	**11**	**x**				**x**		**20**	**30**	**0.67**
**NH**	**12**	**x**					**x**	**10**	**20**	**0.5**
**NH**	**13**	**x**					**x**	**10**	**40**	**0.25**
**NH**	**14**		**x**			**x**	**x**	**0.1**	**25**	**0.004**
**NH**	**15**		**x**			**x**		**5**	**10**	**0.5**
**NH**	**16**		**x**			**x**	**x**	**15**	**20**	**0.75**
**NH**	**17**		**x**				**x**	**5**	**40**	**0.13**
**NH**	**18**	**x**						**25**	**10**	**2.5**
**NH**	**19**		**x**			**x**		**5**	**15**	**0.33**
**NH**	**20**		**x**	**x**		**x**		**10**	**25**	**0.4**
**NH**	**21**		**x**			**x**	**x**	**10**	**20**	**0.5**
**NH**	**22**		**x**			**x**	**x**	**10**	**15**	**0.67**
**NH**	**23**	**x**						**15**	**15**	**1**
**NH**	**24**		**x**			**x**	**x**	**5**	**10**	**0.5**
**NH**	**25**		**x**				**x**	**5**	**15**	**0.33**
**NH**	**26**		**x**			**x**		**20**	**35**	**0.57**
**NH**	**27**	**x**						**10**	**10**	**1**
**NH**	**28**	**x**				**x**		**10**	**20**	**0.5**
**NH**	**29**	**x**						**15**	**25**	**0.6**
**NH**	**30**	**x**				**x**		**10**	**25**	**0.4**
**NH**	**31**		**x**					**10**	**10**	**1**
**NH**	**32**	**x**						**5**	**15**	**0.33**
**NH**	**33**		**x**			**x**		**20**	**20**	**1**
**NH**	**34**		**x**			**x**		**5**	**5**	**1**
**NH**	**35**		**x**			**x**		**5**	**10**	**0.5**
**NH**	**36**	**x**						**15**	**20**	**0.75**
**NH**	**37**	**x**						**20**	**25**	**0.8**
**NH**	**38**	**x**		**x**		**x**		**15**	**15**	**1**
**NH**	**39**		**x**			**x**		**15**	**20**	**0.75**
**NH**	**40**		**x**			**x**	**x**	**20**	**40**	**0.5**
**NH**	**41**		**x**					**10**	**15**	**0.67**
**NH**	**42**		**x**	**x**		**x**		**20**	**15**	**1.33**
**NH**	**43**		**x**					**5**	**10**	**0.5**
**NH**	**44**		**x**	**x**		**x**		**5**	**10**	**0.5**
**NH**	**45**	**x**						**15**	**25**	**0.6**
**NH**	**46**	**x**				**x**		**20**	**15**	**1.33**
**NH**	**47**		**x**					**5**	**10**	**0.5**
**NH**	**48**	**x**				**x**		**30**	**40**	**0.75**
**NH**	**49**		**x**				**x**	**5**	**15**	**0.33**
**NH**	**50**		**x**			**x**	**x**	**15**	**25**	**0.6**
**NH**	**51**	**x**					**x**	**15**	**20**	**0.75**
**NH**	**52**		**x**			**x**	**x**	**10**	**20**	**0.5**
**NH**	**53**	**x**				**x**		**15**	**20**	**0.75**

**PC**	**1**				**x**			**15**	**20**	**0.75**
**PC**	**2**				**x**			**10**	**15**	**0.67**
**PC**	**3**		**x**	**x**				**20**	**10**	**2**
**PC**	**4**		**x**	**x**				**10**	**15**	**0.67**
**PC**	**5**				**x**			**10**	**15**	**0.67**
**PC**	**6**		**x**	**x**						
**PC**	**7**		**x**			**x**	**x**	**15**	**20**	**0.75**
**PH**	**1**	**x**						**40**	**50**	**0.8**
**PH**	**2**		**x**	**x**				**15**	**20**	**0.75**
**PH**	**3**		**x**	**x**				**40**	**50**	**0.8**
**PH**	**4**		**x**					**20**	**25**	**0.8**
**PH**	**5**	**x**						**40**	**45**	**0.88**
**PH**	**6**	**x**						**20**	**30**	**0.67**
**PH**	**7**	**x**				**x**	**x**	**2**	**5**	**0.4**
**PH**	**8**		**x**	**x**				**25**	**15**	**1.67**
**PH**	**9**	**x**						**10**	**15**	**0.67**
**PH**	**10**	**x**						**15**	**40**	**0.38**
**PH**	**11**	**x**						**20**	**30**	**0.67**
**PH**	**12**	**x**						**40**	**20**	**2**
**PH**	**13**	**x**						**20**	**40**	**0.5**
**PH**	**14**	**x**						**25**	**15**	**1.67**
**PH**	**15**	**x**						**30**	**20**	**1.5**
**PH**	**16**		**x**			**x**		**25**	**30**	**0.83**
**PH**	**17**	**x**		**x**				**15**	**15**	**1**
**PH**	**18**	**x**						**25**	**15**	**1.67**
**PH**	**19**		**x**			**x**	**x**	**10**	**20**	**0.5**
**PH**	**20**	**x**						**50**	**15**	**3.33**
**PH**	**21**	**x**						**20**	**5**	**4**
**PH**	**22**	**x**					**x**	**40**	**15**	**2.67**
**PH**	**23**	**x**						**20**	**15**	**1.33**

Figure S4. Overview of lymphocytic infiltration pattern of each case.
